# Combination of Mycorrhizal Symbiosis and Root Grafting Effectively Controls Nematode in Replanted Coffee Soil

**DOI:** 10.3390/plants9050555

**Published:** 2020-04-27

**Authors:** The Trinh Pham, Bach Long Giang, Ngoc Hoi Nguyen, Pham Nguyen Dong Yen, Vo Do Minh Hoang, Bui Thi Lien Ha, Ngoc Thuy Trang Le

**Affiliations:** 1 Department of Science and Technology in DakLak province, 15A Truong Chinh, Buon Ma Thuot City 630000, Vietnam; trinhkhcn@yahoo.com; 2 Faculty of Chemical Engineering and Food Technology, Nguyen Tat Thanh University, 300A Nguyen Tat Thanh, District 4, Ho Chi Minh City 700000, Vietnam; blgiang@ntt.edu.vn; 3 Institute of Applied Materials Science, Vietnam Academy of Science and Technology, Ho Chi Minh City 700000, Vietnam; hoi83bmt@gmail.com (N.H.N.); phamngdongyen@gmail.com (P.N.D.Y.); hvodominh@gmail.com (V.D.M.H.); lienha09@gmail.com (B.T.L.H.); 4 Institute of Research and Development, Duy Tan University, Danang 550000, Vietnam

**Keywords:** coffee, grafting, mycorrhizal symbiosis, nematode management, replanted coffee soil

## Abstract

Replanting for sustainable development is one of the critical missions of the coffee industry in the Daklak province, Vietnam. However, this plan has been faced with many difficulties including poor survival and growth rates due to the low nematode tolerance of young coffee plants in replanted fields. Mycorrhizal symbiosis and grafting have been applied separately but not yet resulted in the expected results of the replanting plan. Whether the combination of them would help managing nematode in the soil and consequently enhance the replanted efficiency is largely unknown. Mycorrhizal symbiosis was applied to *Coffea canephora* plants or/and grafted onto *Coffea liberica* rootstock, which were grown and compared to the untreated ones in both net-house-pots and the replanted plantation. The survival rate, growth indicators and the soil pathogens were monitored during the experimental periods. The combination of grafting and mycorrhiza symbiotic techniques significantly decreased the nematode densities in the replanted soil. As a result, the survival rate and growth indicators of the coffee in the replanted soil treated by the combined technique were better than treated by the two separate techniques. The results suggested that the combination of grafting and mycorrhiza symbiotic techniques would propose a potentially effective *Pratylenchus coffeae* and *Meloidogyne incognita* nematode management in replanted coffee fields in the Daklak province, Vietnam.

## 1. Introduction

Vietnam is the second largest coffee producer and the biggest Robusta exporter in the world. Recently, Vietnam’s coffee area was about 720 thousand ha with the production of more than 1700 thousand tons, of which the Daklak province is the biggest coffee region with 204 thousand ha and 490 thousand tons [1]. Due to the sustainable development of the coffee industry, “Coffee Rejuvenation Strategies in Vietnam” were decided by the government which planned for nearly 28 thousand ha of old, low production and low quality coffee areas to be renewed [2,3,4]. However, the replanted area of coffee in the province has reached a modest figure compared to the plan expectation due to the low survival rate of young coffee trees in replanted fields [5].

Plant-parasitic nematodes, a major limiting factor in crop production areas worldwide, were reported to cause great economic losses to both the coffee farmers and the industry [6]. Scientists have been looking for a variety of tools and methods to control plant-parasitic nematodes that are not only highly effective but also ecofriendly [7,8,9,10]. In Vietnam, due to the long-term monoculture and intensive cultivation of coffee with mainly chemical fertilizers and almost no organic fertilizers, harmful microorganisms are more likely to survive and thrive than beneficial microorganisms. Twenty-one plant-parasitic nematodes species representing 14 genera were recovered from coffee cultivating soil, in which *Pratylenchus coffeae* and *Meloidogyne* spp. were reported to be the predominant genera, which were widely distributed in coffee plantations with 124 and 257 individuals/250 cm^3^, respectively [11]. Nematodes in replanted soils are considered as one of the reasons for the poor result of the Daklak Province Coffee Replanted Plan even though several approaches have been studied and applied to control nematodes in replanted coffee farms such as plowing land after up-rooting and combining with rotation with soil treatments [12].

Mycorrhizal symbiosis and grafting are two separately common horticultural techniques which have been studied and applied in the cultures of many crops [13,14]. Mycorrhiza stimulates coffee tree growth from the nursery stage until the harvesting stage and largely determines the bean yield and quality. Even when soil fertility was reduced and coffee farms were degraded, active infection of mycorrhiza could restore coffee growth. It is also proven that mycorrhiza helps coffee plants to stabilize and prevent nematode attacks [15,16,17]. On the other hand, grafting is a practice where a rootstock is desired which has better characteristics than the commercial variety [18]. Among the recommended rootstocks for coffee, the most common are the Robusta Nemaya variety and the Arabica scion on the Robusta nematode-resistant rootstock, which have been successfully employed in Central America [19,20]. However, the poor compatibility of Robusta grafted onto Excelsa (*Syn. liberica*) rootstocks, which resisted nematodes, was reported from Indonesia [21]. Clear evidence of graft union problems of Robusta grafted onto Excelsa rootstocks was also seen in a field visit in Vietnam. Some coffee farmers have used Excelsa coffee seedlings for rootstocks, but up to 60% losses still occurred due to nematodes since this species was genetically very diverse and seedlings segregated widely for any resistance [22]. Whether the combination of mycorrhizal symbiosis and grafting would help controlling nematodes in the soil and consequently enhance the replanted efficiency is largely unknown.

In this study, it was hypothesized that the combination of grafting and mycorrhizal symbiosis could increase the survival rate and growth of young Robusta coffee trees through controlling nematode densities in the soil. Experiments were carried out with original plants of *C. canephora* as the controls and treated plants to which were applied mycorrhizal symbiosis or/and grafted onto *C. liberica* rootstock in net-house-pots and the replanted plantation. The survival rate, growth indicators and the nematode densities from the controls and the treated plants were monitored and compared during the experimental periods. From these results, appropriate cropping practices could be figured out to control nematodes in replanted coffee fields in the Daklak province, Vietnam.

## 2. Results

### 2.1. Nematode Densities in Soil in Net-House-Pot Experiments

**Figure 1:**
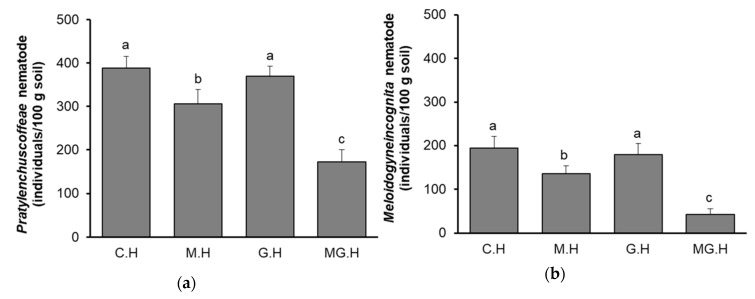
Densities of nematodes after 2 years of cultivating soils in net-house-pot experiments. (**a**) *P. coffeae* density (individuals/100 g soil); (**b**) *M. incognita* density (individuals/100 g soil). C.H, control; M.H, mycorrhizal symbiosis treatment; G.H, grafting treatment; MG.H, combined mycorrhizal symbiosis and grafting treatment. Bars show means ± SD. Bars with the same letters are not statistically different based on the least significant difference at *p* < 0.05.

To evaluate the effect of the combination of mycorrhical symbiosis and grafting on nematode management, *C. canephora* (commonly known as Robusta coffee) plants with mycorrhizal symbiosis or/and grafted onto *C. liberica* rootstock were first grown and compared to the untreated ones after two years of cultivation in net-house-pots. Moreover, the previous coffee surveys in the DakLak province, Vietnam, have revealed a widespread incidence of five nematode genera in Robusta coffee roots, with *Pratylenchus coffeae (P. coffeae)* and *Meloidogyne incognita (M. incognita)* being considered the most important [22]. Therefore, to ascertain the effect of mycorrhiza symbiosis combined with grafting on nematode management, the densities of the two nematodes in the soil after two years testing were determined and are shown in Figure 1.

As shown in Figure 1, the densities of both *P. coffeae* and *M. incognita* of the control (C.H), which was original *C. canephora* without mycorrhizal symbiosis or grafting treatments, were higher than the others treated with mycorrhizal fungi or/and grafting. Independently, the mycorrhizal symbiosis showed better effects on reducing the nematode densities compared to grafting. Soil from the mycorrhizal symbiosis treatment (M.H) had both *P. coffeae* and *M. incognita* densities lower than in the grafted treatment (G.H). No statistically significant differences of nematode densities were recorded between the soil samples from C.H and G.H treatments. Interestingly, the soil from the samples treated with mycorrhizal symbiosis combined with grafting (MG.H) had significant lower nematode densities with 173 individuals/100 g soil for *P. coffeae* (Figure 1a) and 43 individuals/100 g soil for *M. incognita* (Figure 1b) (*p* < 0.05).

### 2.2. Survival Rate and Growth Indicators of Young Robusta Trees in Net-House-Pot Experiments

The brief results of the survival rate and growth indicators of the young Robusta trees in the net-house-pots are presented in Table 1. After two years of cultivation in net-house-pots, the survival rates and especially the growth indicators of all the treatments were higher than the control. Mycorrhizal symbiosis and grafting techniques separately slightly increased the growth indicators of the treated young Robusta, however, the differences were not statistically significant at *p* < 0.05. In addition, the young Robusta trees strongly improved their survival rate and growth indicators when they were treated with the combination of the mycorrhizal symbiosis and the grafting. This result was according to the nematode density results explained before.

### 2.3. Nematode Densities in Soil in Replanted Field Experiments

**Figure 2:**
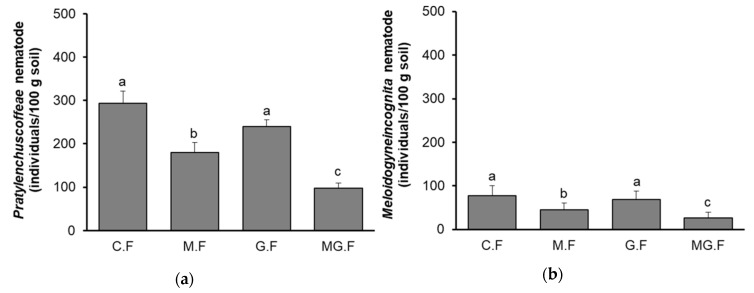
Densities of nematodes after 2 years of cultivating soils in replanted field experiments. (**a**) *P. coffeae* density (individuals/100 g soil); (**b**) *M. incognita* density (individuals/100 g soil). C.F, control; M.F, mycorrhizal symbiosis treatment; G.F, grafting treatment; MG.F, combined mycorrhizal symbiosis and grafting treatment. Bars show means ± SD. Bars with the same letters are not statistically different based on the least significant difference at *p* < 0.05.

To confirm the efficiency of the combined technique, the same treatments included the control (C.F), which was original *C. canephora* with neither mycorrhizal symbiosis nor grafting treatments, *C. canephora* trees with applied mycorrhizal symbiosis (M.F), *C. canephora* trees grafted onto *C. liberica* rootstock (G.F) and *C. canephora* trees treated with the combined technique (MG.F) which were grown and compared after two years of cultivation on replanted coffee fields. The densities of *P. coffeae* and *M. incognita* in the soil after two years testing were determined and are shown in Figure 2.

In general, the results from the replanted field experiments were similar with the results from net-house-pot experiments. The densities of *P. coffeae* and *M. incognita* in the replanted coffee soil were 294 and 78 individuals/100 g soil, respectively, from the control (C.F), which was higher than in the soil from the M.F, G.F and MG.F treatments. Mycorrhizal symbiosis again indicated better effects on reducing nematode densities compared to the grafting due to the densities of the two nematodes in the soil from the M.F treatment being lower than that in the soil from the G.H treatment. However, there was no statistically significant difference of nematode densities between the control and grafting treatments. Similar to the net-house-pot results, the combination of mycorrhizal symbiosis and grafting helped strongly decrease the nematode densities in the soil to 98 individuals/100 g soil for *P. coffeae* (Figure 2a) and 27 individuals/100 g soil for *M. incognita* (Figure 2b) (*p* < 0.05).

### 2.4. Survival Rates and Growth Indicators of Young Robusta Trees in Replanted Field Experiments

Similar to the results of the experiments carried out in net-house-pots, the treated young Robusta trees had higher survival rates than the control. These results of the two monitored years were in accordance with each other and are shown in Table 2. Generally, the ratio of tree deaths in the first year was higher than in the second year. Mycorrhizal symbiosis or/and grafting significantly improved the survival rates of young coffee trees during the two year cultivation in the replanted fields. Even though the mycorrhizal symbiosis seemed to enhance the survival rate more effectively than the grafting, the difference was not statistically significant. The combination of the two techniques considerably raised young Robusta viability from 60.5% for the C.F to 93.7% for the MG.F in the second year (*p* < 0.05).

The growth indicators of young Robusta trees were monitored during two years of cultivation in a replanted coffee plantation and the brief results are shown in Table 3. For tree height, stump diameter and canopy diameter indicators, grafting seemed to create a better improvement compared to the mycorrhizal symbiosis. There was no difference in the number of first branch pairs between the M.F and the G.F (*p* < 0.05). The MG.F treatment had the highest growth indicators after two years of cultivation in the replanted fields.

## 3. Discussion

The current study examined the effects of two cropping techniques including mycorrhizal symbiosis and grafting on nematode management to improve the replanting efficiency of young Robusta trees in replanted fields in the Daklak province, Vietnam. Three key findings arose from the study: Firstly, separately applying mycorrhizal symbiosis and grafting *C. canephora* scion onto *C. liberica* rootstock both slightly decreased nematode densities in the replanted coffee soil in the net-house-pots and plantations, consequently improving the viability and growth of young coffee trees. Secondly, even though the mycorrhizal symbiosis treatment indicated a better improvement on controlling nematodes in replanted coffee soil than the grafting treatment, the survival rate of the young Robusta trees treated by these two techniques were not statistically different (*p* < 0.05). Thirdly, the combination of the grafting and the mycorrhizal symbiosis that was recorded greatly decreased the nematode densities, leading to the highest efficiency on the seedling young Robusta coffee trees in the replanted fields.

In general, the treatments which resulted in high nematode densities in the soil would correspondingly lead to the low survival and growth rates of coffee plants. This result strengthens the assumption that nematodes are one of the reasons for low replanting efficiency. The most common coffee variety used as the rootstock for grafting is Robusta [23]. However, in order to improve the root development and the tree growth of Robusta, the most popular coffee variety in the Daklak province, Liberica is expected to be a good rootstock. The experimental results in net-house-pots as well as in the field of this study proved this expectation to be true. This result can be explained by Liberica’s ability to endure extreme conditions and resist pests which has been reported to be better than that of Robusta [24]. On the other hand, mycorrhiza fungi have been used as an environmentally friendly agent to manage nematodes [25,26,27]. The two antagonistic fungi in the mycorrhiza commercial product, *Trichoderma harzianum* and *Trichoderma viride*, were reported to especially reduce *M. incognita* and *P. coffeae* [28,29]. The mycorrhizal symbiosis technique has also been applied to improve the growth and development of plants thanks to its ability to support nitrogen fixation as well as to absorb nutrients in the soil of the root system [30]. The study results once again confirmed this when both the M.H and M.F treatments had lower nematode densities in the soil, and enjoyed higher survival rates and growth indicators compared to the C.H and C.F, respectively.

It is interesting to be aware that even though the mycorrhizal symbiosis treatment indicated a better improvement on decreasing nematodes in replanted coffee soil than the grafting treatment, the survival rates of young Robusta trees separately treated by these two techniques (M.F and G.F) were not statistically different (*p* < 0.05). This can be explained by the main mechanisms of mycorrhiza fungi and grafting rootstock. Mycorrhiza fungi directly effects on the pathogen, by competiting for space or nutrients [31]. Hence, mycorrhiza fungi and nematodes most probably depend on common resources such as host photosynthate, nutrition, infection-site and space within the plant root for their survival and multiplication; hence competition can occur for these resources between nematodes and mycorrhizal fungi [32]. Meanwhile the main characteristics of the grafting technique are a better root development and pest tolerance, thus generating conditions so that the crop does not stop its metabolic activities such as the absorption of water and nutrients [18]. From these literatures, it can be assumed that the high survival rates of young Robusta trees in the M.H and M.F treatments were due to the nematode competition of mycorrhiza, while in the G.H and G.F treatments they were due to the nematode tolerance of the grafting rootstock.

The combination of the two techniques greatly decreased the nematode densities, leading to the highest benefit for the seedling young Robusta coffee trees in the replanted fields among the treatments. This combined technique took advantage of the benefits of each component technique. As a result, the efficiency of the nematode management in the replanted coffee fields was boosted by not only the decreasing nematode densities in the soil but also by the increasing nematode tolerance of trees. The combination of the mycorrhizal symbiosis and grafting techniques to manage nematodes has been studied and obtained positive results in several plants such as cucumber, plum and tomato [33,34,35]. The successful result of this combined technique in the nematode management would contribute as a useful reference to crop production in general and coffee production in particular. In the current research, the nematode densities in the soil as well as the survival rate and growth rate of young Robusta coffee trees were recorded for the first two years (the basic construction period). The effect of the combination of the mycorrhiza symbiotic and grafting techniques on the yield as well as on the quality of the coffee beans should also be further investigated.

## 4. Materials and Methods

### 4.1. Field Sight

The experiment was carried out in the Daklak province, located in the Central-Highlands in Vietnam. Its geographic coordinates are 12°40′ N and 108°03′ E with an average elevation of about 400 to 800 m. Daklak province has a temperate climate, it is warm year-round and rarely changes yearly. Ferralsols (mainly red basaltic soil) is the main soil group in Daklak and occupies 23.7% area of the province [1].

### 4.2. Materials

The original plants of *C. canephora* and the grafting plants of *C. canephora* Scion onto the *C. liberica* Rootstock at 6-month age were supplied by the Daklak province plant nursery. Mycorrhizal symbiosis was performed by using a commercial mycorrhiza product, supplied by the Vietnam Soils and Fertilizers Research Institute, with three different species of mycorrhiza fungi including *Glomus* spp., *Gigaspora* spp., *Acaulospora* spp. and other nutrients and beneficial microorganisms (nitrogen fixing, phosphate solubilizing, and disease fungal antagonizing (*T. harzianum* and *T. viride* species)). The spore density of the used mycorrhiza product was >100 spores/g inoculants. A solution of mycorrhiza product was prepared at the concentration of 500 g/litter before inoculating. The nematode populations of *P. coffeae* and *M. incognita* were isolated from the research sites in the Daklak province following the centrifugal flotation method [36] and then cultured on carrot callus tissue [37]. Ten males and ten females of each population were used to prepare the nematode morphological collection slides following the De Grisse method [38]. The nematode samples were fixed, observed under electronic microscopes and identified by the classification method of Castillo and Volvlas [11,39].

### 4.3. Evaluation of the Effect of Mycorrhizal Symbiosis and Grafting Techniques on Nematode Densities in Net-House-Pots

The experiment was carried out from November 2015 to June 2018 in a randomized complete block design in the net-house of the Vietnam Soils and Fertilizers Research Institute as follows: treatments = 4; treatment size = 15; replication = 3 (Table 4). The ambient condition of the net-house was similar to the local climate, whose temperature was 22–27 °C and humidity was 70–90%. The soil was collected from the replanted coffee plantations in the Cu M’gar district, Daklak province, and transported to the experimental net-house and then sterilized to kill all pathogens in the soil before filling into polythene bags and pots in the net-house for the experiments. The soil amounts in each polythene bag and pot were 2 kg and 5 kg, respectively. Nematode and mycorrhiza inoculations were separately performed as follows: the mycorrhiza were added by directly irrigating mycorrhiza product solution into soil. The first mycorrhizal inoculation into the nursery was carried out at a dose of 100 g/plant, and then the inoculated plants (in polythene bags) were left for 5 days before being transferred to pots. After 3 months, the second mycorrhizal inoculation into the pots was performed with the amount of 100 g/tree. After transferring the coffee plants from the polythene bags to pots, 5000 nematode individuals were added into every pot. Ten days later, the second nematode inoculation into the pots was conducted with the same dose. The individual ratio of *P. coffeae* and *M. incognita* in each 5000-individual population was chosen as 2:1 due to fact that the initial densities of *P. coffeae* and *M. incognita* were found to be 112 ± 11 and 31 ± 7 individuals/100g soil, respectively (individual ratio was approximately 2). This meant that there were 10,000 nematode individuals and 200 g mycorrhizal products (approximately 20,000 mycorrhiza spores) inoculated in each pot (tree). In other words, there were approximate 134 *P. coffeae* individuals, 66 *M. incognita* individuals and 400 mycorrhiza spores in 100 g of pot soil. The amount of nitrogen-phosphorus-potassium (NPK) fertilizer was calculated and applied according to the following local procedure: 12 tons of manure; 60N; 90P_2_O_5_; 40K_2_O/ha (first year); 90N; 90P_2_O_5_; 90K_2_O/ha (second year). The survival rate and growth indicators such as tree height, stump diameter, canopy diameter and number of first level branch pairs were monitored every 6 months over 2 years. The densities of nematodes in the soil were determined after 2 years of cultivation.

### 4.4. Evaluation of the Effect of Mycorrhizal Symbiosis and Grafting Techniques on Nematode Densities in Replanted Coffee Fields

The experiment was carried out from November 2016 to June 2019 in a randomized complete block design in a replanted coffee plantation in the Cu M’gar district, Daklak province, as follows: treatments = 4; replication = 4; block area = 250 m^2^; planting hole size = 3 m × 3 m (Table 5). The soil in the plantation was red brown soil developed on basalt. After uprooting the old coffee trees, the soil was applied lime and dried for 6 months during the dry season. In the process of tillage, the remaining coffee roots were picked up. The mycorrhiza product was applied twice. The first inoculation into the nursery was carried out at a dose of 100 g/plant. After 4 months of growing the coffee trees in the replanted soil, the second inoculation was performed with the amount of 100 g/tree. The amount of NPK fertilizer was applied similarly to the net-house experiments. The survival rate and growth indicators as well as the nematode densities were monitored similarly to the net-house experiments.

### 4.5. Data Collection and Analysis

The survival rate of the coffee plants was determined by the following formulation:

(1) $$\mathrm{Survival}\;\mathrm{rate}\;\left(\%\right)=\frac{B-A}{A}\times 100\%$$

where: A was the total of dead plants and plants with yellowing leaves and B was the total experimental plants.

The data was aggregated and analyzed using the Statistic Analyze Software of Excel.

## 5. Conclusions

The separate treatments, namely the mycorrhizal symbiosis and the grafting of *C. canephora* scion onto the *C. liberica* rootstock, slightly decreased the nematode densities in the replanted coffee soil in the net-house-pots and in the replanted fields, and consequently improved the viability and growth of the young coffee trees. The mycorrhizal symbiosis treatment indicated a better improvement on controlling the nematode density in replanted coffee soil than the grafting treatment but the survival rates of the young Robusta trees treated by these two techniques were similar. The combination of grafting and the mycorrhizal symbiosis was recorded to greatly decrease the nematode densities, leading to the highest efficiency on the seedling young Robusta coffee trees in the replanted fields. The efficiency of nematode management in the replanted coffee fields was boosted not only by the decreasing nematode densities in the soil but also by the increasing nematode tolerance of the trees. This recommends that the combined technique of grafting and mycorrhizal symbiosis would be an appropriate cropping practice to manage nematodes in replanted coffee fields in the Daklak province, Vietnam. In the future, how this combination effects on yield and the quality of coffee beans should also be further investigated.

## Figures and Tables

**Table 1:** Survival rate and the growth indicators of the young Robusta trees after 2 years of cultivation in net-house-pots.

Treatment	Survival Rate (%)	Growth Indicators			
		Tree Height (cm)	Stump Diameter (cm)	Canopy Diameter (cm)	No. of 1st Branch Pairs
**C.H**	66.7 ^a^	130.2 ^a^	2.37 ^a^	135.0 ^a^	8.75 ^a^
M.H	78.2 ^b^	138.3 ^a^	2.40 ^a^	138.0 ^a^	9.82 ^a^
G.H	72.3 ^b^	138.2 ^a^	2.54 ^a^	133.5 ^a^	9.43 ^a^
MG.H	100 ^c^	147.4 ^b^	2.73 ^b^	145.9 ^b^	10.63 ^b^
CV (%)	2.69	4.16	3.41	4.25	4.26
LSD_0.05_	3.523	4.274	0.458	6.58	2.366

C.H, control; M.H, mycorrhizal symbiosis treatment; G.H, grafting treatment; MG.H, combined mycorrhizal symbiosis and grafting treatment. CV, Coefficient of variation. LSD, Least significant difference. Means with the same letters are not statistically different based on the least significant difference at *p* < 0.05.

**Table 2:** Survival rate of the young Robusta coffee trees after 2 years of cultivation in the replanted fields.

Treatment	Survival Rate (%)	
	First Year	Second Year
C.F	73.8 ^a^	60.5 ^a^
M.F	80.5 ^b^	71.2 ^b^
G.F	79.5 ^b^	69.5 ^b^
MG.F	95.8 ^c^	93.7 ^c^
CV (%)	4.37	4.02
LSD_0.05_	3.152	2.781

C.F, control; M.F, mycorrhizal symbiosis treatment; G.F, grafting treatment; MG.F, combined mycorrhizal symbiosis and grafting treatment. CV, Coefficient of variation. LSD, Least significant difference. Means with the same letters are not statistically different based on the least significant difference at *p* < 0.05.

**Table 3:** Growth indicators of the young Robusta coffee trees after 2 years of cultivation in the replanted fields.

Treatment	Tree Height (cm)	Stump Diameter (cm)	Canopy Diameter (cm)	Number of 1st Branch Pairs
C.F	112.6 ^a^	2.41 ^a^	126.7 ^a^	10.5 ^a^
M.F	116.8 ^a^	2.58 ^a^	129.4 ^a^	10.4 ^a^
G.F	128.6 ^b^	3.72 ^b^	142.7 ^b^	10.4 ^a^
MG.F	128.9 ^b^	3.84 ^b^	143.2 ^b^	10.9 ^b^

C.F, control; M.F, mycorrhizal symbiosis treatment; G.F, grafting treatment; MG.F, combined mycorrhizal symbiosis and grafting treatment. Means with the same letters are not statistically different based on the least significant difference at *p* < 0.05.

**Table 4:** Treatments for evaluating the effect of the mycorrhizal symbiosis and grafting techniques on the nematode densities in the net-house-pots.

Treatment	Description
C.H	Control (neither mycorrhizal symbiosis nor grafting) in net-house-pot
M.H	Mycorrhizal symbiosis treatment in net-house-pot
G.H	Grafting treatment in net-house-pot
MG.H	Combined mycorrhizal symbiosis and grafting in net-house-pot

C.H, control; M.H, mycorrhizal symbiosis treatment; G.H, grafting treatment; MG.H, combined mycorrhizal symbiosis and grafting treatment.

**Table 5:** Treatments for evaluating the effect of the mycorrhizal symbiosis and grafting techniques on the nematode densities in the replanted fields.

Treatment	Description
C.F	Control (neither mycorrhizal symbiosis nor grafting) in replanted fields
M.F	Mycorrhizal symbiosis treatment in replanted fields
G.F	Grafting treatment in replanted fields
MG.F	Combined mycorrhizal symbiosis and grafting in replanted fields

C.F, control; M.F, mycorrhizal symbiosis treatment; G.F, grafting treatment; MG.F, combined mycorrhizal symbiosis and grafting treatment.
